# Piperidinium *N*-(ferrocenylcarbon­yl)glycinate

**DOI:** 10.1107/S1600536810041747

**Published:** 2010-11-06

**Authors:** Petr Štěpnička, Martin Zábranský, Ivana Císařová

**Affiliations:** a Department of Inorganic Chemistry, Faculty of Science, Charles University in Prague, Hlavova 2030, 12840 Prague 2, Czech Republic

## Abstract

The title compound, (C_5_H_12_N)[Fe(C_5_H_5_)(C_8_H_7_NO_3_)], resulting from neutralization of *N*-(ferrocenylcarbon­yl)glycine with piperidine, is built up from discrete ions that assemble into sheets via the combination of conventional and weak hydrogen bonds. The key repeating unit is constituted by two piperidium cations and two carboxylate anions that assemble into a centrosymmetric array *via* conventional and bifurcated N—H⋯O hydrogen bonds. The aggregates thus formed are further interlinked by N—H⋯O interactions and supportive C—H⋯O contacts into layers oriented parallel to the *bc* plane.

## Related literature

For an overview of bioorganometallic chemistry of ferrocene, see: Štěpnička (2008 ▶). For the first synthesis of *N*-(ferrocenylcarbon­yl)glycine, see: Schlögl (1957 ▶) and for its use in the preparation of 2-ferrocenyl-5(4*H*)oxazolone and its transition metal complexes, see: Bauer *et al.* (1999 ▶). An alternative preparative route was described by Kraatz *et al.* (1997 ▶). For the crystal structures of methyl *N*-(ferrocenylcarbon­yl)glycinate and *tert*-butyl *N*-[1′-(diphenyl­phosphino)ferrocene-1-carbon­yl]glycinate, see: Gallagher *et al.* (1999 ▶) and Tauchman *et al.* (2009 ▶), respectively. The structure of another related compound, 1,1′-bis­{*N*-(carb­oxy­methyl­ene)carbamo­yl}ferro­cene, was reported by Appoh *et al.* (2004 ▶).  
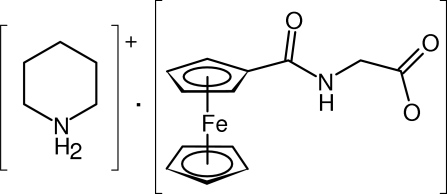


## Experimental

### 

#### Crystal data

(C_5_H_12_N)[Fe(C_5_H_5_)(C_8_H_7_NO_3_)]
*M* *_r_* = 372.24
Monoclinic, 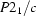

*a* = 13.9055 (4) Å
*b* = 7.6150 (2) Å
*c* = 16.5968 (5) Å
β = 105.780 (2)°
*V* = 1691.21 (8) Å^3^
*Z* = 4
Mo *K*α radiation
μ = 0.91 mm^−1^
*T* = 150 K
0.25 × 0.22 × 0.15 mm

#### Data collection

Nonius KappaCCD diffractometer
25843 measured reflections
3456 independent reflections
2960 reflections with *I* > 2σ(*I*)
*R*_int_ = 0.044

#### Refinement

*R*[*F* ^2^ > 2σ(*F* ^2^)] = 0.031
*wR*(*F* ^2^) = 0.076
*S* = 1.11
3456 reflections
217 parameters
H-atom parameters constrained
Δρ_max_ = 0.27 e Å^−3^
Δρ_min_ = −0.29 e Å^−3^

### 

Data collection: *COLLECT* (Nonius, 2000 ▶); cell refinement: *HKL* *SCALEPACK* (Otwinowski & Minor, 1997 ▶); data reduction: *HKL* *DENZO* (Otwinowski & Minor, 1997 ▶) and *SCALEPACK*; program(s) used to solve structure: *SIR97* (Altomare *et al.*, 1999 ▶); program(s) used to refine structure: *SHELXL97* (Sheldrick, 2008 ▶); molecular graphics: *PLATON* (Spek, 2009 ▶); software used to prepare material for publication: *PLATON*.

## Supplementary Material

Crystal structure: contains datablocks I, global. DOI: 10.1107/S1600536810041747/dn2612sup1.cif

Structure factors: contains datablocks I. DOI: 10.1107/S1600536810041747/dn2612Isup2.hkl

Additional supplementary materials:  crystallographic information; 3D view; checkCIF report

## Figures and Tables

**Table 1:** Hydrogen-bond geometry (Å, °)

*D*—H⋯*A*	*D*—H	H⋯*A*	*D*⋯*A*	*D*—H⋯*A*
N2—H2*N*⋯O2	0.91	1.86	2.764 (2)	172
N2—H3*N*⋯O3^i^	1.00	1.76	2.749 (2)	172
N2—H3*N*⋯O2^i^	1.00	2.60	3.325 (2)	130
N1—H1*N*⋯O3^ii^	0.89	2.04	2.908 (2)	166
C5—H5⋯O3^ii^	0.93	2.56	3.366 (3)	146
C12—H12*B*⋯O1^ii^	0.97	2.39	3.307 (3)	158

Symmetry codes: (i) 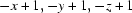; (ii) 
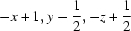
.

**Fig. 1.**
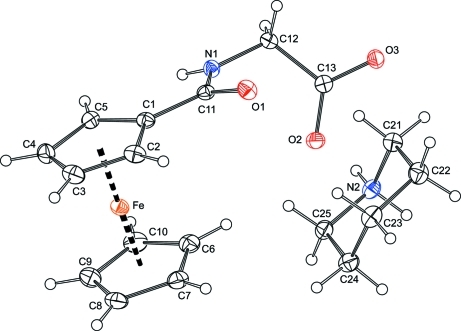
View of the ions constituting the structure of the title compound showing the atom numbering scheme and displacement ellipsoids for the non-H atoms at the 30% probability level (N.B. Mutual orientation of the ions corresponds with that encountered in the crystal).

**Fig. 2.**
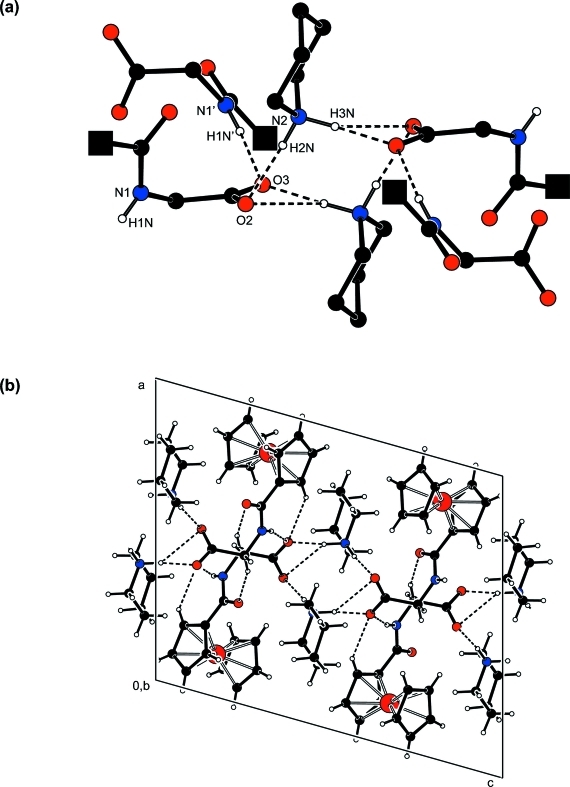
(a) View of the hydrogen-bonded aggregate in the crystal structure of the title compound, showing the H-bonds as dashed lines. For clarity, only NH protons are shown and the bulky ferrocenyl groups are replaced with filled black squares. (c) View of the unit cell along the crystallographic b axis.

## supplementary crystallographic information

### Comment

ferrocenylcarbonyl derivatives (amides) prepared from amino acids and peptides received considerable research attention in the recent past, mainly as precursors to redox-labelled biomolecules and as model compounds for studies focusing on stereochemistry and H-bonding interactions in peptides (Štěpnička, 2008). *N*-(ferrocenylcarbonyl)glycine as the first ferrocenylated amino acid derivative was reported already in 1957 (Schlögl, 1957). It was further used in the preparation of 2-ferrocenyl-5(4*H*)oxazolone and its complexes with transition metals (Bauer *et al.*, 1999) and further studied as a redox-responsive reagent for inorganic anions (Gallagher *et al.*, 1999).

Thus, piperidinium *N*-(ferrocenylcarbonyl)glycinate was obtained by acid-base reaction *N*-(ferrocenylcarbonyl)glycine with piperidine and isolated as a yellow, air-stable crystalline solid. Its molecular structure as determined by X-ray diffraction analysis (Figure 1) is rather unexceptional. The geometry of the ferrocene unit is regular, showing a practically negligible variation in the Fe—C bond lengths (2.029 (19)–2.051 (2) Å). Accordingly, the distance of the iron atom to cyclopentadienyl ring centroids are quite similar (1.6429 (10) and 1.6450 (10) Å for the rings C(1–5) and C(6–10), respectively), and the dihedral angle of the least-squares cyclopentadienyl planes is only 2.28 (13) °.

The geometry of the glycinamide moiety is similar to those reported previously for methyl *N*-(ferrocenylcarbonyl)glycinate (Gallagher *et al.*, 1999) or *t*-butyl *N*-[1'-(diphenylphosphino)ferrocene-1-carbonyl]glycinate (Tauchman *et al.*, 2009). A difference is seen in the parameters describing the terminal carboxy group, which is deprotonated (unlike the reference compounds) and shows balanced C—O distance due to delocalization (C13—O2 = 1.255 (2) Å, C13—O3 = 1.265 (2) Å, O2—C13—O3 = 124.25 (17) °). The amide plane, {C11, O1, N1}, is rotated with respect to its bonding cyclopentadienyl ring C(1–5) by only 8.9 (2) °. As a results, the two moieties remain conjugated, which is reflected by a relative shortening of the connecting C1—C11 bond (1.486 (3) Å). Similar bond lengths (1.483 (2) and 1.491 (2) Å) were observed for two monoclinic polymorphs of carbamoylferrocene, where the amide and cyclopentadienyl planes are rotated by *ca* 10 ° (Štěpnička *et al.*, 2010). The glycine moeity extends below the parent cyclopentadienyl ring at the dihedral angle C11—N1—C12—C13 of 59.3 (1) °. Finally, the piperidinium cation assumes an envelope conformation with τ = 178.1 (2) ° and puckering amplitude *Q* = 0.572 (2) Å (*N.B.* ideal chair requires τ = 0 or 180 °). There is, however, noticeable some departure from the regular geometry since the C(21/25)—N bond lengths are slightly shorter (*ca* 2%) than the remaining in-ring distances (C—C = 1.513 (3)–1.525 (3) Å).

The crystal packing of the title compound is dominated by intermolecular hydrogen bonding interaction. The ions constituting the structure assemble pairwise around inversion centers by means of N—H···O hydrogen bonds from both NH protons at the piperidinium cation to adjacent carboxylate O atoms O2 and O3 as H-bond acceptors (Figure 2a; for parameters, see Table 1). An additional contact, N2—H3N···O2 has a rather unfavourable geometry and probably reflects an enforced proximity of the atoms involved. The four-membered centrosymmetric assemblies thus formed are interconnected by N—H···O hydrogen bonds between *amide* NH and carboxylate O3 and further by supportive C—H···O interactions (Figure 2a, Table 1) into layers oriented parallel to the *bc* plane (Figure 2b).

### Experimental

*N*-(ferrocenylcarbonyl)glycine was prepared in analogy with the literature (Kraatz *et al.*, 1997; Bauer *et al.*, 1999; Appoh *et al.*, 2004) as follows. Ferrocenecarboxylic acid and glycine methyl ester hydrochloride were reacted in dichloromethane in the presence peptide coupling agents (1-ethyl-3-[3-(dimethylamino)propyl]carbodiimide and 1-hydroxy-1*H*-1,2,3-benzotriazole) and triethylamine, which *in situ* converts glycine methyl ester hydrochloride to the respective free base. The resulting methyl *N*-(ferrocenylcarbonyl)glycinate was isolated by column chromatography (silica, dichloromethane–methanol 10:1). This ester was saponified with NaOH in refluxing water–dioxane (1:1). Subsequent acidification H_3_PO_4_ afforded free acid, which was isolated and purified by column chromatography on silica using dichloromethane–methanol 5:1 as the eluent.

Yellow crystals of the title compounds were obtained by mixing equimolar amounts of piperidine and *N*-(ferrocenylcarbonyl)glycine (50 µmol each) in dry methanol (*ca* 1 ml) and subsequent crystallization by a slow diffusion of diethyl ether vapours. Analysis calcd. for C_18_N_24_FeN_2_O_3_: C, 58.08; H, 6.50; N, 7.53%. Found: C, 57.73; H, 6.65; N, 7.36%.

### Refinement

H-atoms residing on the carbon atoms were included in their calculated positions and treated as riding atoms with *U*_iso_(H) = 1.2 *U*_eq_(C). Those biding to the N and O atoms were identified on the difference electron density maps and refined as described above.

### Crystal data

**Table:**

(C_5_H_12_N)[Fe(C_5_H_5_)(C_8_H_7_NO_3_)]	*F*(000) = 784
*M**_r_* = 372.24	*D*_x_ = 1.462 Mg m^−^^3^
Monoclinic, *P*2_1_/*c*	Mo *K*α radiation, λ = 0.71073 Å
Hall symbol: -P 2ybc	Cell parameters from 3696 reflections
*a* = 13.9055 (4) Å	θ = 1.0–26.4°
*b* = 7.6150 (2) Å	µ = 0.91 mm^−^^1^
*c* = 16.5968 (5) Å	*T* = 150 K
β = 105.780 (2)°	Plate, yellow
*V* = 1691.21 (8) Å^3^	0.25 × 0.22 × 0.15 mm
*Z* = 4	

### Data collection

**Table:**

Nonius KappaCCD diffractometer	2960 reflections with *I* > 2σ(*I*)
Radiation source: fine-focus sealed tube	*R*_int_ = 0.044
horizontally mounted graphite crystal	θ_max_ = 26.4°, θ_min_ = 1.5°
Detector resolution: 9.091 pixels mm^-1^	*h* = −17→17
ω and π scans to fill the Ewald sphere	*k* = −9→9
25843 measured reflections	*l* = −20→20
3456 independent reflections	

### Refinement

**Table:**

Refinement on *F*^2^	Primary atom site location: structure-invariant direct methods
Least-squares matrix: full	Secondary atom site location: difference Fourier map
*R*[*F*^2^ > 2σ(*F*^2^)] = 0.031	H-atom parameters constrained
*wR*(*F*^2^) = 0.076	*w* = 1/[σ^2^(*F*_o_^2^) + (0.0234*P*)^2^ + 1.3244*P*] where *P* = (*F*_o_^2^ + 2*F*_c_^2^)/3
*S* = 1.11	(Δ/σ)_max_ < 0.001
3456 reflections	Δρ_max_ = 0.27 e Å^−^^3^
217 parameters	Δρ_min_ = −0.29 e Å^−^^3^
0 restraints	

### Special details

**Table:**

Geometry. All e.s.d.'s (except the e.s.d. in the dihedral angle between two least-squares planes) are estimated using the full covariance matrix. The cell e.s.d.'s are taken into account individually in the estimation of e.s.d.'s in distances, angles and torsion angles; correlations between e.s.d.'s in cell parameters are only used when they are defined by crystal symmetry. An approximate (isotropic) treatment of cell e.s.d.'s is used for estimating e.s.d.'s involving least-squares planes.
Refinement. Refinement of *F*^2^ against all diffractions. The weighted *R*-factor *wR* and goodness of fit *S* are based on *F*^2^, conventional *R*-factors *R* are based on *F*, with *F* set to zero for negative *F*^2^. The threshold expression of *F*^2^ > 2σ(*F*^2^) is used only for calculating *R*-factors(gt) *etc*. and is not relevant to the choice of reflections for refinement. *R*-factors based on *F*^2^ are statistically about twice as large as those based on *F*, and *R*- factors based on all data will be even larger.

### Fractional atomic coordinates and isotropic or equivalent isotropic displacement parameters (Å^2^)

**Table:**

	*x*	*y*	*z*	*U*_iso_*/*U*_eq_	
Fe	0.14161 (2)	0.21877 (4)	0.174028 (17)	0.02423 (9)	
O1	0.33180 (10)	0.60519 (18)	0.23818 (9)	0.0292 (3)	
O2	0.45779 (10)	0.33934 (18)	0.36777 (8)	0.0276 (3)	
O3	0.58097 (10)	0.53347 (18)	0.38131 (8)	0.0288 (3)	
N1	0.40631 (11)	0.3748 (2)	0.19374 (10)	0.0231 (3)	
H1N	0.3988	0.2730	0.1668	0.028*	
C1	0.22784 (14)	0.4080 (3)	0.14255 (12)	0.0238 (4)	
C2	0.13442 (14)	0.4816 (3)	0.14607 (13)	0.0286 (4)	
H2	0.1258	0.5733	0.1805	0.034*	
C3	0.05759 (16)	0.3900 (3)	0.08792 (14)	0.0339 (5)	
H3	−0.0106	0.4109	0.0776	0.041*	
C4	0.10200 (16)	0.2611 (3)	0.04804 (13)	0.0338 (5)	
H4	0.0681	0.1831	0.0070	0.041*	
C5	0.20758 (15)	0.2717 (3)	0.08169 (12)	0.0275 (4)	
H5	0.2548	0.2020	0.0666	0.033*	
C6	0.22092 (16)	0.1253 (3)	0.28749 (13)	0.0323 (5)	
H6	0.2831	0.1657	0.3186	0.039*	
C7	0.12645 (16)	0.1899 (3)	0.29199 (13)	0.0331 (5)	
H7	0.1160	0.2795	0.3268	0.040*	
C8	0.05104 (17)	0.0937 (3)	0.23426 (14)	0.0364 (5)	
H8	−0.0175	0.1092	0.2244	0.044*	
C9	0.09846 (17)	−0.0297 (3)	0.19440 (15)	0.0369 (5)	
H9	0.0664	−0.1098	0.1536	0.044*	
C10	0.20368 (17)	−0.0108 (3)	0.22739 (14)	0.0352 (5)	
H10	0.2524	−0.0763	0.2121	0.042*	
C11	0.32579 (14)	0.4699 (2)	0.19603 (11)	0.0225 (4)	
C12	0.50438 (14)	0.4274 (3)	0.24432 (12)	0.0230 (4)	
H12A	0.5197	0.5426	0.2262	0.028*	
H12B	0.5536	0.3458	0.2346	0.028*	
C13	0.51381 (14)	0.4337 (2)	0.33808 (12)	0.0230 (4)	
N2	0.37021 (12)	0.5724 (2)	0.45421 (10)	0.0283 (4)	
H2N	0.4048	0.5013	0.4277	0.034*	
H3N	0.3928	0.5410	0.5148	0.034*	
C21	0.39265 (16)	0.7595 (3)	0.44159 (13)	0.0329 (5)	
H21A	0.3741	0.7862	0.3822	0.039*	
H21B	0.4638	0.7805	0.4637	0.039*	
C22	0.33498 (16)	0.8779 (3)	0.48587 (14)	0.0343 (5)	
H22A	0.3467	0.9997	0.4743	0.041*	
H22B	0.3590	0.8599	0.5459	0.041*	
C23	0.22331 (16)	0.8399 (3)	0.45705 (13)	0.0326 (5)	
H23A	0.1974	0.8728	0.3987	0.039*	
H23B	0.1891	0.9095	0.4896	0.039*	
C24	0.20298 (16)	0.6455 (3)	0.46742 (13)	0.0325 (5)	
H24A	0.2212	0.6159	0.5265	0.039*	
H24B	0.1322	0.6221	0.4446	0.039*	
C25	0.26207 (15)	0.5325 (3)	0.42294 (12)	0.0297 (4)	
H25A	0.2507	0.4095	0.4324	0.036*	
H25B	0.2397	0.5544	0.3632	0.036*	

### Atomic displacement parameters (Å^2^)

**Table:**

	*U*^11^	*U*^22^	*U*^33^	*U*^12^	*U*^13^	*U*^23^
Fe	0.02232 (15)	0.02174 (15)	0.02877 (16)	0.00023 (12)	0.00721 (11)	0.00277 (12)
O1	0.0284 (7)	0.0236 (7)	0.0364 (8)	0.0000 (6)	0.0102 (6)	−0.0050 (6)
O2	0.0302 (7)	0.0273 (7)	0.0278 (7)	−0.0035 (6)	0.0119 (6)	0.0000 (6)
O3	0.0303 (7)	0.0277 (8)	0.0252 (7)	−0.0055 (6)	0.0021 (6)	0.0017 (6)
N1	0.0234 (8)	0.0221 (8)	0.0238 (8)	−0.0004 (7)	0.0064 (7)	−0.0030 (7)
C1	0.0241 (10)	0.0215 (9)	0.0258 (10)	0.0010 (8)	0.0070 (8)	0.0055 (8)
C2	0.0266 (10)	0.0224 (10)	0.0379 (11)	0.0047 (8)	0.0106 (9)	0.0078 (9)
C3	0.0241 (10)	0.0334 (12)	0.0417 (12)	0.0023 (9)	0.0046 (9)	0.0138 (10)
C4	0.0303 (11)	0.0381 (12)	0.0288 (10)	−0.0063 (9)	0.0011 (9)	0.0030 (9)
C5	0.0280 (10)	0.0300 (11)	0.0249 (10)	−0.0005 (9)	0.0082 (8)	0.0007 (8)
C6	0.0330 (11)	0.0314 (12)	0.0312 (11)	0.0008 (9)	0.0064 (9)	0.0112 (9)
C7	0.0390 (12)	0.0317 (12)	0.0314 (11)	−0.0013 (10)	0.0143 (9)	0.0036 (9)
C8	0.0324 (12)	0.0377 (12)	0.0421 (12)	−0.0059 (10)	0.0152 (10)	0.0077 (10)
C9	0.0468 (13)	0.0231 (11)	0.0408 (13)	−0.0088 (10)	0.0118 (11)	0.0024 (10)
C10	0.0417 (12)	0.0241 (11)	0.0412 (13)	0.0075 (9)	0.0135 (10)	0.0098 (9)
C11	0.0263 (10)	0.0197 (9)	0.0231 (9)	−0.0006 (8)	0.0094 (8)	0.0028 (8)
C12	0.0217 (9)	0.0210 (10)	0.0272 (10)	−0.0006 (8)	0.0081 (8)	−0.0001 (8)
C13	0.0238 (9)	0.0194 (9)	0.0260 (10)	0.0042 (8)	0.0071 (8)	0.0010 (8)
N2	0.0316 (9)	0.0311 (9)	0.0234 (8)	0.0044 (8)	0.0096 (7)	−0.0014 (7)
C21	0.0315 (11)	0.0366 (12)	0.0311 (11)	−0.0042 (9)	0.0098 (9)	−0.0012 (9)
C22	0.0400 (12)	0.0276 (11)	0.0343 (11)	0.0012 (10)	0.0086 (10)	−0.0025 (9)
C23	0.0351 (11)	0.0318 (11)	0.0306 (11)	0.0091 (9)	0.0084 (9)	0.0020 (9)
C24	0.0307 (11)	0.0376 (12)	0.0302 (11)	0.0030 (9)	0.0102 (9)	0.0037 (9)
C25	0.0329 (11)	0.0304 (11)	0.0248 (10)	−0.0014 (9)	0.0061 (9)	0.0004 (9)

### Geometric parameters (Å, °)

**Table:**

Fe—C5	2.0290 (19)	C7—C8	1.417 (3)
Fe—C1	2.0316 (19)	C7—H7	0.9300
Fe—C6	2.034 (2)	C8—C9	1.412 (3)
Fe—C7	2.037 (2)	C8—H8	0.9300
Fe—C4	2.038 (2)	C9—C10	1.423 (3)
Fe—C9	2.041 (2)	C9—H9	0.9300
Fe—C10	2.042 (2)	C10—H10	0.9300
Fe—C8	2.044 (2)	C12—C13	1.526 (3)
Fe—C3	2.049 (2)	C12—H12A	0.9700
Fe—C2	2.051 (2)	C12—H12B	0.9700
O1—C11	1.235 (2)	N2—C25	1.483 (3)
O2—C13	1.255 (2)	N2—C21	1.485 (3)
O3—C13	1.265 (2)	N2—H2N	0.9128
N1—C11	1.342 (2)	N2—H3N	0.9980
N1—C12	1.450 (2)	C21—C22	1.522 (3)
N1—H1N	0.8865	C21—H21A	0.9700
C1—C5	1.422 (3)	C21—H21B	0.9700
C1—C2	1.430 (3)	C22—C23	1.523 (3)
C1—C11	1.486 (3)	C22—H22A	0.9700
C2—C3	1.414 (3)	C22—H22B	0.9700
C2—H2	0.9300	C23—C24	1.525 (3)
C3—C4	1.416 (3)	C23—H23A	0.9700
C3—H3	0.9300	C23—H23B	0.9700
C4—C5	1.424 (3)	C24—C25	1.513 (3)
C4—H4	0.9300	C24—H24A	0.9700
C5—H5	0.9300	C24—H24B	0.9700
C6—C10	1.413 (3)	C25—H25A	0.9700
C6—C7	1.424 (3)	C25—H25B	0.9700
C6—H6	0.9300		
			
C5—Fe—C1	41.00 (8)	C10—C6—C7	108.0 (2)
C5—Fe—C6	121.73 (8)	C10—C6—Fe	70.00 (12)
C1—Fe—C6	106.07 (8)	C7—C6—Fe	69.64 (12)
C5—Fe—C7	158.99 (9)	C10—C6—H6	126.0
C1—Fe—C7	123.11 (8)	C7—C6—H6	126.0
C6—Fe—C7	40.93 (9)	Fe—C6—H6	125.9
C5—Fe—C4	40.99 (8)	C8—C7—C6	108.0 (2)
C1—Fe—C4	68.76 (8)	C8—C7—Fe	69.93 (12)
C6—Fe—C4	158.79 (9)	C6—C7—Fe	69.43 (12)
C7—Fe—C4	158.97 (9)	C8—C7—H7	126.0
C5—Fe—C9	121.53 (9)	C6—C7—H7	126.0
C1—Fe—C9	157.05 (9)	Fe—C7—H7	126.2
C6—Fe—C9	68.42 (9)	C9—C8—C7	107.9 (2)
C7—Fe—C9	68.21 (9)	C9—C8—Fe	69.68 (12)
C4—Fe—C9	107.89 (9)	C7—C8—Fe	69.44 (12)
C5—Fe—C10	105.85 (9)	C9—C8—H8	126.1
C1—Fe—C10	120.62 (8)	C7—C8—H8	126.1
C6—Fe—C10	40.57 (9)	Fe—C8—H8	126.4
C7—Fe—C10	68.45 (9)	C8—C9—C10	108.3 (2)
C4—Fe—C10	123.02 (9)	C8—C9—Fe	69.88 (12)
C9—Fe—C10	40.80 (9)	C10—C9—Fe	69.63 (12)
C5—Fe—C8	158.01 (9)	C8—C9—H9	125.8
C1—Fe—C8	160.30 (9)	C10—C9—H9	125.8
C6—Fe—C8	68.62 (9)	Fe—C9—H9	126.2
C7—Fe—C8	40.63 (9)	C6—C10—C9	107.8 (2)
C4—Fe—C8	123.03 (9)	C6—C10—Fe	69.43 (12)
C9—Fe—C8	40.44 (9)	C9—C10—Fe	69.57 (12)
C10—Fe—C8	68.46 (9)	C6—C10—H10	126.1
C5—Fe—C3	68.66 (8)	C9—C10—H10	126.1
C1—Fe—C3	68.52 (8)	Fe—C10—H10	126.5
C6—Fe—C3	158.55 (9)	O1—C11—N1	122.50 (18)
C7—Fe—C3	123.40 (9)	O1—C11—C1	120.83 (17)
C4—Fe—C3	40.54 (9)	N1—C11—C1	116.63 (17)
C9—Fe—C3	124.61 (9)	N1—C12—C13	113.93 (15)
C10—Fe—C3	160.19 (9)	N1—C12—H12A	108.8
C8—Fe—C3	109.10 (9)	C13—C12—H12A	108.8
C5—Fe—C2	68.87 (8)	N1—C12—H12B	108.8
C1—Fe—C2	41.02 (7)	C13—C12—H12B	108.8
C6—Fe—C2	122.11 (9)	H12A—C12—H12B	107.7
C7—Fe—C2	108.14 (9)	O2—C13—O3	124.25 (17)
C4—Fe—C2	68.31 (9)	O2—C13—C12	119.45 (17)
C9—Fe—C2	160.71 (9)	O3—C13—C12	116.28 (16)
C10—Fe—C2	157.30 (9)	C25—N2—C21	112.28 (16)
C8—Fe—C2	124.52 (9)	C25—N2—H2N	109.1
C3—Fe—C2	40.34 (8)	C21—N2—H2N	110.0
C11—N1—C12	119.69 (16)	C25—N2—H3N	108.4
C11—N1—H1N	120.1	C21—N2—H3N	110.5
C12—N1—H1N	119.7	H2N—N2—H3N	106.3
C5—C1—C2	107.95 (18)	N2—C21—C22	109.99 (17)
C5—C1—C11	128.91 (18)	N2—C21—H21A	109.7
C2—C1—C11	123.13 (18)	C22—C21—H21A	109.7
C5—C1—Fe	69.40 (11)	N2—C21—H21B	109.7
C2—C1—Fe	70.21 (11)	C22—C21—H21B	109.7
C11—C1—Fe	125.49 (13)	H21A—C21—H21B	108.2
C3—C2—C1	107.76 (19)	C21—C22—C23	111.20 (18)
C3—C2—Fe	69.78 (12)	C21—C22—H22A	109.4
C1—C2—Fe	68.77 (11)	C23—C22—H22A	109.4
C3—C2—H2	126.1	C21—C22—H22B	109.4
C1—C2—H2	126.1	C23—C22—H22B	109.4
Fe—C2—H2	126.9	H22A—C22—H22B	108.0
C2—C3—C4	108.44 (18)	C22—C23—C24	110.71 (18)
C2—C3—Fe	69.89 (11)	C22—C23—H23A	109.5
C4—C3—Fe	69.30 (12)	C24—C23—H23A	109.5
C2—C3—H3	125.8	C22—C23—H23B	109.5
C4—C3—H3	125.8	C24—C23—H23B	109.5
Fe—C3—H3	126.6	H23A—C23—H23B	108.1
C3—C4—C5	108.16 (19)	C25—C24—C23	110.77 (17)
C3—C4—Fe	70.16 (12)	C25—C24—H24A	109.5
C5—C4—Fe	69.17 (11)	C23—C24—H24A	109.5
C3—C4—H4	125.9	C25—C24—H24B	109.5
C5—C4—H4	125.9	C23—C24—H24B	109.5
Fe—C4—H4	126.3	H24A—C24—H24B	108.1
C1—C5—C4	107.69 (18)	N2—C25—C24	110.26 (17)
C1—C5—Fe	69.60 (11)	N2—C25—H25A	109.6
C4—C5—Fe	69.85 (12)	C24—C25—H25A	109.6
C1—C5—H5	126.2	N2—C25—H25B	109.6
C4—C5—H5	126.2	C24—C25—H25B	109.6
Fe—C5—H5	126.0	H25A—C25—H25B	108.1

### Hydrogen-bond geometry (Å, °)

**Table:**

*D*—H···*A*	*D*—H	H···*A*	*D*···*A*	*D*—H···*A*
N2—H2N···O2	0.91	1.86	2.764 (2)	172
N2—H3N···O3^i^	1.00	1.76	2.749 (2)	172
N2—H3N···O2^i^	1.00	2.60	3.325 (2)	130
N1—H1N···O3^ii^	0.89	2.04	2.908 (2)	166
C5—H5···O3^ii^	0.93	2.56	3.366 (3)	146
C12—H12B···O1^ii^	0.97	2.39	3.307 (3)	158

Symmetry codes: (i) −*x*+1, −*y*+1, −*z*+1; (ii) −*x*+1, *y*−1/2, −*z*+1/2.
